# Bipyrazone: a new HPPD-inhibiting herbicide in wheat

**DOI:** 10.1038/s41598-020-62116-6

**Published:** 2020-03-26

**Authors:** Hengzhi Wang, Weitang Liu, Tao Jin, Xuegang Peng, Lele Zhang, Jinxin Wang

**Affiliations:** 1 College of Plant Protection, Shandong Agricultural University, Tai’an, 271018 Shandong P.R. China; 2 Key Laboratory of Pesticide Toxicology and Application Technique, College of Plant Protection, Shandong Agricultural University, Tai’an, 271018 Shandong P.R. China; 3 Qingdao Kingagroot Chemical Compound Co., Ltd., Qingdao, 266000 Shandong P.R. China; 4 Administration Bureau of the Yellow River Delta National Nature Reserve, Dongying, 257091 Shandong P.R. China

**Keywords:** Drug safety, Toxicology

## Abstract

Bipyrazone, 1,3-dimethyl-4-(2-(methylsulfonyl)-4-(trifluoromethyl) benzoyl)-1H-pyrazol-5-yl 1,3-dimethyl-1H-pyrazole- 4-carboxylate, is a 4-hydroxyphenylpyaunate dioxygenase (HPPD)-inhibiting herbicide. Greenhouse and field experiments were conducted to explore the potential of post-emergence (POST) application of bipyrazone in wheat fields in China. In the greenhouse study, bipyrazone at 10 and 20 g active ingredient (a.i.) ha^−1^ effectively controlled *Descurainia sophia* L., *Capsella bursa-pastoris* (L.) Medic., *Lithospermum arvense* L. and *Myosoton aquaticum* L. Whereas, all tested 16 wheat cultivars showed high degree of tolerance to bipyrazone at 375 and 750 g a.i. ha^−1^. In a dose-response experiment carried on the Shannong 6 wheat cultivar and five weed biotypes, bipyrazone was safe to the wheat cultivar, and *C. bursa-pastoris*, *M. aquaticum* and *D. sophia* were sensitive to this herbicide. The selectivity index (SI) between the Shannong 6 and weeds ranged from 34 to 39. The field experiments confirmed that a mixture of bipyrazone and fluroxypyr-mepthyl is practical for controlling broadleaf weeds, and bipyrazone applied alone at 30 to 40 g a.i. ha^−1^ can also provide satisfactory control of sensitive broadleaf weeds. These findings suggest that bipyrazone POST application has good potential for broadleaf weed management in wheat fields.

## Introduction

Wheat (*Triticum aestivum* L.) is one of the most important food crops in China, with 24.1 million hectares planted and 126 million tons produced every year^1^. It is the staple food for millions of people, particularly in Northern China. Weeds have a high impact on wheat yield and quality by competing for water, light and nutrients^2^, causing 30% production loss every year^3^. Chemical technology has played an important role in weed control in the wheat fields of China due to the many advantages of herbicides. However, with continuous and excessive use, the problem of herbicide resistance in weeds is increasingly significant. More and more broadleaf weeds have been reported to have evolved resistance to commonly used herbicides in many parts of China. For example, 28 populations of *C. bursa-pastoris* from different locations in Henan Province, one of the major wheat-producing areas in China, have evolved resistance to the ALS inhibitor tribenuron-methyl^4^. Some *M. aquaticum* populations have evolved resistance to several ALS inhibitors^5^ and the resistance ratio of *D. sophia* to tribenuron-methyl was up to 1594 compared to the susceptible population^6^. Herbicide resistant weeds are an important constraint on agricultural development and threatens grain production worldwide^7,8^. Therefore, herbicides with different modes of action to tribenuron-methyl, one of the main herbicides used for selective broadleaf weed control in wheat fields in China, are badly needed to solve the problem of broadleaf weed resistance.

**Figure 1:**
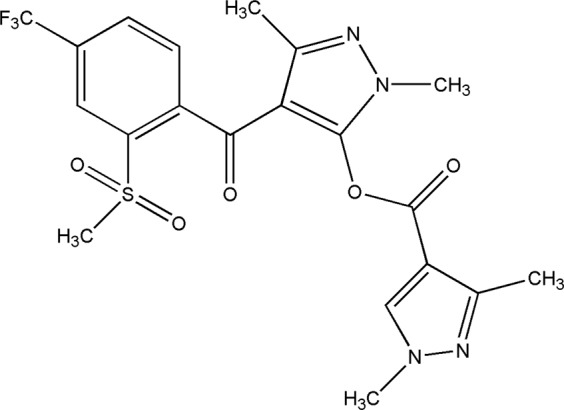
Structure of bipyrazone used in the experiments. 1,3-dimethyl-4-(2-(methylsulfonyl)-4-(trifluoromethyl)benzoyl)-1H-pyrazol-5-yl, 3-dimethyl-1H-pyrazole- 4-carboxylate.

Bipyrazone, 1,3-dimethyl-4-(2-(methylsulfonyl)-4-(trifluoromethyl) benzoyl)-1H-pyrazol-5-yl 1,3-dimethyl-1H-pyrazole- 4-carboxylate (Patent Number: ZL2014102275916; CAS: 1622908-18-2) (Fig. 1), is a new herbicide developed by Qingdao Kingagroot Chemical Compound Co., Ltd. It is from the newest generation of 4-hydroxyphenylpyaunate dioxygenase (HPPD) inhibitors, and may be a new candidate for solving the current broadleaf weed resistance problem in wheat fields in China. HPPD-inhibiting herbicides have been used for selective weed control since 1980, when pyrazolate (used for rice) was on the market, but before its HPPD mode of action was recognized^9^. They inhibit the HPPD enzyme, an essential enzyme that converts 4-hydroxyphenylpyruvic acid (4-HPPA) to homogentisic acid (HGA), as well as affecting the biosynthesis of plastoquinone (PQ) and α-tocopherol^10,11^. PQ is a critical factor of phytoene desaturase enzyme (PDS) in the biosynthesis of carotenoid, protecting photosystems against photooxidative damage^12^. Moreover, tocopherol could quench and scavenge reactive oxygen species (ROS) such as ^1^O_2_ and OH- radicals^12–14^. The excessive ROS result in oxidative degradation of chlorophyll and photosynthetic membranes in the growing shoot tissues of susceptible plants^15^. The combination of these effects results in bleaching and death of the susceptible plant.

In the last four decades, a number of HPPD inhibitors have been developed for weed control^16^, but none have been used in wheat fields in China^17^. The objectives of this study were (1) to determine the efficacy of bipyrazone against 14 common broadleaf weeds in China, and the tolerance of 16 wheat cultivars in greenhouse conditions; (2) to identify the selectivity of bipyrazone between one common Chinese wheat cultivar and five biotypes of three common weed species in wheat fields in China; and (3) to identify and evaluate efficient application rates of bipyrazone and its mixture with fluroxypyr-mepthyl under field conditions.

## Materials and Methods

### Weeds and Wheat Cultivars

The species used in the weed control spectrum study (Table 1) were collected from uncultivated areas in Tai’an Shandong (36.21°N,117.09°E) or Shouxian Anhui (32.56°N,116.81°E) in eastern China. The wheat cultivars used in the tolerance study are presented in Table 2. These cultivars were provided by the College of Agriculture, Shandong Agricultural University. The biotypes of *C. bursa-pastoris* (HN05) and *D. sophia* (JS16) with target-site-resistance (TSR) to tribenuron-methyl used in the selectivity index study were preserved from our previous study^4,18^.

**Table 1:** Dry weight inhibition of different weeds treated with bipyrazone relative to the untreated control in the greenhouse study 21 days after treatment.

Weeds	Classification	Dry weight inhibition (SE)^a^	
		10 g a.i. ha^−1^	20 g a.i. ha^−1^
		%	
Shepherd’s purse [*Capsella bursa-pastoris* (L.) Medic.]	Dicotyledon	77 (0.6)	93 (0.4) *
Flixweed [*Descurainia sophia* L.]	Dicotyledon	77 (1.5)	92 (1.1) *
Water starwort [*Myosoton aquaticum* L.]	Dicotyledon	60 (0.5)	90 (0.4) *
Corn gromwell [*Lithospermum arvense* L.]	Dicotyledon	60 (1.6)	85 (0.9) *
Sun spurge [*Euphorbia helioscopia* L.]	Dicotyledon	27 (0.7)	36 (1.8) *
Carolina geranium [*Geranium carolinianum* L.]	Dicotyledon	11 (0.4)	30 (0.7) *
Common vetch [*Vicia sativa* L.]	Dicotyledon	9 (1.3)	26 (1.1) *
Catchweed bedstraw [*Galium aparine* L.]	Dicotyledon	8 (1.0)	19 (0.7) *
Asian copperleaf [*Acalypha australis* L.]	Dicotyledon	3 (0.9)	11 (0.9) *
Persian speedwell [*Veronica persica* Poir.]	Dicotyledon	6 (2.9)	9 (1.6) NS
Water foxtail [*Alopecurus aequalis* Sobol.]	Monocotyledon	4 (1.5)	7 (2.1) NS
Goosegrass [*Eleusine indica* (L.) Gaertn.]	Monocotyledon	5 (2.7)	6 (3.2) NS
Wild oat [*Avena fatua* L.]	Monocotyledon	5 (2.7)	6 (2.2) NS
Italian Ryegrass [*Lolium multiflorum* Lamk.]	Monocotyledon	0	0

^a^*significant differences between the two rates at 0.05 level according to Fisher’s protected LSD test; NS, not significant; SE, standard error, n = 8.

**Table 2:** Dry weight inhibition of different wheat cultivars treated with bipyrazone POST application relative to the untreated control in the greenhouse study 21 days after treatment.

Wheat cultivars	Dry weight reduction (SE)		Wheat cultivars	Dry weight reduction (SE)	
	375 g a.i. ha^−1^	750 g a.i. ha^−1^		375 g a.i. ha^−1^	750 g a.i. ha^−1^
	%			%	
Shannong 6	3 (0.6)	11 (0.3) *	Jimai 22	1 (0.4)	7 (0.3) *
Jimai 20	3 ((0.3)	14 (0.3) *	Shannong 16	4 (0.4)	13 (0.2) *
Weimai 8	2 (0.3)	8 (0.3) *	Zongmai 1	4 (0.2)	14 (0.4) *
Shandongheima 1	3 (0.5)	11 (0.3) *	Zhengmai 101	2 (0.3)	9 (0.4) *
Shannong 20	1 (0.3)	7 (0.5) *	Zhengmai 314	3 (0.3)	13 (0.3) *
Shannong 22	3 (0.4)	11 (0.4) *	Zhoumai 22	2 (0.4)	10 (0.7) *
Linmai 4	3 (0.2)	11 (0.2) *	Wennong 14	0	6 (0.2) *
Liangxing 77	4 (0.3)	13 (0.4) *	Tainong 18	3 (0.5)	12 (0.2) *

^a^*significant differences between the two rates at 0.05 level according to Fisher’s protected LSD test; NS, not significant; SE, standard error, n = 8.

### Herbicide Formulation

96% purity bipyrazone (Qingdao Kingagroot Chemical Compound Co., Ltd., Qingdao, China) and 95% purity tribenuron-methyl (Shandong Weifang Rainbow Chemical Co., Ltd, Weifang, China) was dissolved in a suitable volume of acetone to obtain the stock solution, then diluted with 0.1% mass fraction of Tween 80 (Solarbio Life Sciences, Beijing, China) to the rates needed for the greenhouse study. 10% bipyrazone oil dispersion (OD) (Qingdao Kingagroot Chemical Compound Co., Ltd., Qingdao, China), 75% tribenuron-methyl water dispersible granule (WDG) (DuPont Company, Shanghai, China) and 28.8% fluroxypyr-mepthyl emulsifiable concentrate (EC) (Shandong Binnong Technology Company, Binzhou, China) were dissolved and diluted with water to the rates needed for the field experiments.

### Greenhouse Experiment Design

All the greenhouse experiments were conducted from October 2015 to May 2016 in a controlled greenhouse (natural lighting, daytime temperature 20~25 °C and night temperature 15~20 °C, 75% relative humidity) in the Arboretum of Shandong Agricultural University (36.21°N,117.13°E). Before planting, the wheats and weeds were geminated in a petri dish with two layers of wet filter paper or 1% (m/v) plant agar in an illumination chamber (25/15 °C, 12/12 h day/night) (Model RXZ, Ningbojiangnan Instrument Factory, Ningbo, China). As the radicle became visible, nine wheat seeds or twelve weed seeds were sown below the soil surface in plastic pots (16-cm diameter x 13-cm height). The soil had a pH of 7.67, organic matter of 15.79 g kg^−1^ and 14.28-2.0-23.15 g kg^−1^ of N P K, and was passed through a 3-mm sieve. Before herbicide application, the wheat seedlings were thinned to six evenly-sized plants, and weed seedlings were thinned to an appropriate number of plants per pot according to the size of weed. The pots were watered at the bottom, when needed, to maintain the moisture. The seedlings were treated with herbicides at the 3- to 4-leaf stage using a research track sprayer (Model ASS-4, National Agricultural Information Engineering and Technology Center of China) with a Teejet 9503EVS flat-fan nozzle that delivered a 450 L ha^−1^ spray of solution at a spray pressure of 275 kPa. Twenty-one days after treatment (DAT), the shoots were harvested, oven dried at 80 °C for 72 h and the dry weights recorded. All the experiments in the greenhouse were arranged in a randomized complete block design with four replicates and conducted at least twice.

#### Weed Control Spectrum

Fourteen species commonly infesting wheat fields in China were selected to conduct the weed control spectrum study. The POST application of bipyrazone at 10 and 20 g a.i. ha^−1^ was conducted and an untreated control (0.1% mass fraction of Tween 80) was established for each weed species. Other experimental conditions were the same as described in the greenhouse experimental design.

#### Wheat Cultivar Tolerance

Sixteen leading wheat cultivars planted in Northern China were selected to conduct the tolerance experiment. The POST application of bipyrazone at 375 and 750 g a.i. ha^−1^ was conducted and an untreated control (0.1% mass fraction of Tween 80) was established for each wheat cultivar. Other experimental conditions were the same as described in the greenhouse experiment design.

#### Selectivity Index (SI)

The selectivity index is the ratio between the application rate that caused 10% growth reduction in the crop and the rate that caused 90% growth reduction in the weed^19^. Based on the results of the weed control spectrum and wheat cultivar tolerance experiments, three common and troublesome broadleaf weeds *C. bursa-pastoris*, *M. aquaticum* and *D. Sophia* were selected, along with Shannong 6, one of the most popular wheat cultivars in Northern China, in order to study the SI. To determine the sensitivity of herbicide resistant weeds to bipyrazone, the SI between Shannong 6 and the biotypes of *C. bursa-pastoris* and *D. sophia* with TSR to tribenuron-methyl were also determined. Wheat (Shannong 6) was treated with POST application at 0 (0.1% mass fraction of Tween 80), 750, 1500, 3000, 4500, and 6000 g a.i. ha^−1^ of bipyrazone, and *C. bursa-pastoris* (R and susceptible (S) biotypes), *D. sophia* (R and S biotypes) and *M. aquaticum* were treated with POST application at 0 (0.1% mass fraction of Tween 80), 1.25, 2.5, 5, 10 and 20 g a.i. ha^−1^. Other experimental conditions were the same as described in the greenhouse experiment design.

### Field Experiment

In 2017 and 2018, field experiments were conducted twice at farm fields (36.17°N, 117.16°E, loam with 1.7% organic matter and a pH of 7.1) in Tai’an, Shandong, China to determine the weed control efficacy of bipyrazone, tribenuron-methyl, fluroxypyr-mepthyl and the mixtures of bipyrazone with fluroxypyr-mepthyl against broadleaf weeds in winter wheat fields. The test field was heavily infested with *C. bursa-pastoris*, *D. sophia* and *G. aparine* and the tribenuron-methyl has failed to control *C. bursa-pastoris* for several years. The wheat (Jimai 20) was sown in a drill by field equipment on 12 October, 2016 and 20 October, 2017. The weed population in the test field in 2017 and 2018 during the POST application was about 28 to 30 plants m^−2^ for *C. bursa-pastoris*, 12 to 14 plants m^−2^ for *D. sophia*, 20 to 26 plants m^−2^ for *G. aparine* and basically no grassy weeds.

**Figure 2:**
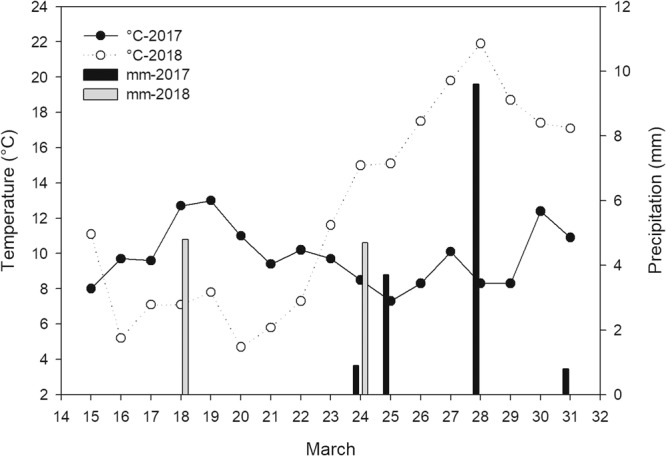
Daily average temperature and precipitation at the experimental site at Tai’an, Shandong, China after several days of treatment in 2017 and 2018. POST application was conducted at Mar 15 in 2017 and Mar 21 in 2018.

Field experiments were designed as randomized complete blocks with four replicates each of 20 m^2^ (5 m × 4 m). The 12 treatments are presented in Table 3 including four rates of bipyrazone, a single rate of tribenuron-methyl, a single rate of fluroxypyr-mepthyl, four rates of a mixture of bipyrazone and fluroxypyr-mepthyl, a hand weeded control and no treatment. The POST application was made on 15 March, 2017 (sunny, −1.7 to 15.5 °C, breeze) and 21 March, 2018 (sunny, 5.8 to 12.9 °C, light breeze) when wheat was at 3-leaf to tillering stages and weeds were at 2- to 5-leaf stages. A total spray volume of 450 L ha^−1^ was applied with a backpack sprayer (Bellspray Inc., Opelousa, LA) equipped with a single 8002 VS nozzle (Teejet Technologies, Wheaton, IL), and wheat production followed the local common farming practice. The daily air temperature and precipitation at the experimental site after several days of treatment are presented in Fig. 2.

**Table 3:** The herbicides, their formulation types and rates in the field experiment.

Treatment number	Herbicides^a^	Rate (g a. i. ha^-1^)
1	10% bipyrazone OD	10.0
2	10% bipyrazone OD	20.0
3	10% bipyrazone OD	30.0
4	10% bipyrazone OD	40.0
5	75% tribenuron-methyl WDG	22.5
6	28.8% fluroxypyr-mepthyl EC	160.0
7	10% bipyrazone OD + 28.8% fluroxypyr-mepthyl EC	30.0 + 90.0
8	10% bipyrazone OD + 28.8% fluroxypyr-mepthyl EC	30.0 + 120.0
9	10% bipyrazone OD + 28.8% fluroxypyr-mepthyl EC	40.0 + 120.0
10	10% bipyrazone OD + 28.8% fluroxypyr-mepthyl EC	40.0 + 160.0
11	Hand weeding	—
12	No treatment	—

^a^OD, oil dispersion; WDG, water dispersible granule; EC, emulsifiable concentrate.

The weed survey was conducted five times in total at 5, 10, 15, 20 and 40 DAT, and a visual estimate was conducted at 5, 10 and 15 DAT. To calculate weed population reduction, all weeds were separated by species, counted and recorded from three 0.33 m^2^ quadrats for each plot at 20 DAT. To calculate weed population and biomass reduction, the shoots of all weed plants were cut from three 0.33 m^2^ quadrats for each plot at 40 DAT, and all weeds were separated by species, counted, oven dried at 80 °C for 72 h and dry weight recorded. The reduction was calculated according to Eq. 1:

1 $${\rm{Reduction}}( \% )=[1\,-\,{\rm{TP}}/({\rm{STP}}/4)]\times 100$$

where TP is weed number or biomass in the treated plot, and STP is the sum of the number or biomass of the four no treatment plots.

The visual wheat damage was evaluated at 5, 10, 20, 40 DAT and harvesting date. Wheat grain yields (adjusted to 13% moisture) were determined by hand harvesting 5 m^2^ of each plot and are expressed on a per-hectare basis.

### Data analysis

The data from repeated greenhouse study were analysed by analysis of variance (ANOVA) using SPSS (Version 17.0; IBM Corporation, Armonk, NY), and the data were pooled because of the non-significant difference between repeated experiments at the 5% significance level. The means were separated using Fisher’s protected least-significant difference (LSD) at the 5% significance level.

The dose-response curves were acquired through non-linear regression analysis using the four-parameter log-logistic response Eq. 2^20^:

2 $${\rm{y}}={\rm{C}}+({\rm{D}}-{\rm{C}})/\{1+\exp [{\rm{b}}(\log ({\rm{x}})\,-\,\log ({{\rm{GR}}}_{50}))]\}$$

where C is the lower limit of response, D is the upper limit of response, x is the herbicide rate, y is the percentage of dry weight residue, GR_50_ is the dose causing 50% growth inhibition and *b* is the slope of the curve around the GR_50_. Graphs were created by SigmaPlot software (Version 13.0; Systat Software Inc., CA, USA) with the dry weight means (% of residual) and standard errors.

According to the regression parameters, the GR_10_ and GR_50_ values for the wheat and GR_50_ and GR_90_ for the weeds were calculated based on Eq. 2^21^. The SIs of bipyrazone were calculated as Eq. 3:

3 $${{\rm{SI}}}_{(10;90)}={{\rm{GR}}}_{10({\rm{wheat}})}/{{\rm{GR}}}_{90({\rm{weed}})}$$

Where GR_10_ equals a 10% growth reduction on Shannong 6 and GR_90_ equals a 90% growth reduction on the trial weeds.

The data from the field experiments were tested for homogeneity of variances and normality of distribution, and subjected to arcsine square root transformation as needed before analysis. All data were subjected to ANOVA, and the means were separated using Fisher’s protected LSD test at 5% significance level. The data were presented as untransformed means, and analysed separately by year because the weather conditions were different each year.

## Results

### Weed control spectrum

At 10 g a.i. ha^−1^, the POST application of bipyrazone was most effective (60%~79% of dry weight inhibition) against four broadleaf weeds out of all weeds tested in the study; these were *D. sophia*, *C. bursa-pastoris*, *L. arvense* and *M. aquaticum* (Table 1). When the rate was increased to 20 g a.i. ha^−1^, the POST application of bipyrazone showed greater efficacy (85%~93% of dry weight inhibition) against the same four broadleaf weeds (Table 1). However, some weed species, especially grassy weed species, were not susceptible to bipyrazone, e.g., *Veronica persica* Poir., *Acalypha australis* L., *Alopecurus aequalis* Sobol., *Eleusine indica* (L.) Gaertn., *Lolium multiflorum* Lamk. and *Avena fatua* L. (Table 1).

### Wheat cultivar tolerance

The POST application of bipyrazone was safe for all 16 tested wheat cultivars under greenhouse conditions and no bleaching symptoms were observed during the whole experiment. The dry weight inhibition of all 16 tested wheat cultivars was below 5% when bipyrazone was applied POST at 375 g a.i. ha^−1^, and below 15% when applied POST at 750 g a.i. ha^−1^ (Table 2).

### Selectivity index

**Figure 3:**
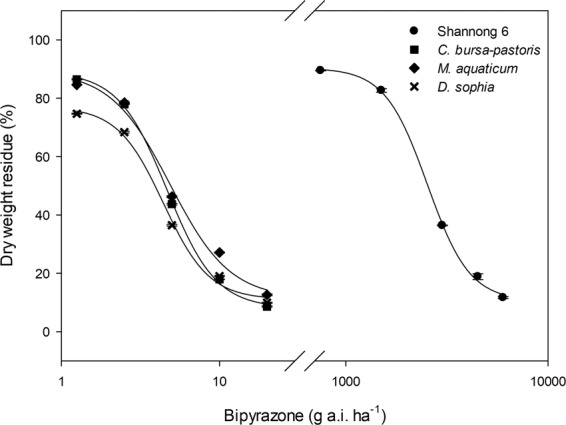
Percentage of dry weight residue of *C. bursa-pastoris*, *M. aquaticum* and *D. sophia* in response to increasing bipyrazone rates in greenhouse study 21 days after treatment (DAT) based on nonlinear regression fit to a four parameter log–logistic curve model: $y=C+(D-C)/\{1+\exp [b(\log (x)-\,\log \,(G{R}_{50}))]\}$.

The tolerance of Shannong 6 wheat to bipyrazone applied POST was much higher than that of weeds, and *D. sophia* was more sensitive to bipyrazone applied POST than *C. bursa-pastoris* and *M. aquaticum* (Fig. 3). The SI of bipyrazone POST application between Shannong 6 wheat and five biotypes of three weed species were from 34 to 39 (Table 4). Moreover, there was no significant difference in sensitivity (GR_50_ value) to bipyrazone between susceptible and resistant biotypes of *C. bursa-pastoris* and *M. aquaticum* (Table 4).

**Table 4:** Parameters of the four-parameter log-logistic equation used to calculate application rates of bipyrazone causing 10% (GR_10_) and 50% (GR_50_) growth reduction of Shannong 6, and 50% and 90% (GR_90_) growth reduction of five biotypes of weeds, and the selectivity index (SI) between Shannong 6 and five biotypes of weeds in the greenhouse study 21 days after treatment.

Trial plants^a^	Regression parameters (SE)^b^			GR _10_	GR_50_ (SE)^c^	GR_90_	SI^d^
	C	D	b				
				g a.i. ha^−1^			
Shannong 6	10.6 (2.6)	90.4 (2.3)	−4.1 (0.6)	688.55	2531.4 (98.8) a	—	—
*C. bursa-pastoris*	8.1 ((2.5)	89.0 (2.9)	−2.8 (0.4)	—	4.7 (0.2) c	18.66	37
*M. aquaticum*	11.4 (9.2)	89.4 (9.7)	−2.3 (1.1)	—	4.9 (0.9) bc	17.86	39
*D. sophia*	11.1 (4.3)	77.1 (5.5)	−3.0 (1.0)	—	4.4 (0.5) d	19.98	34
R- *C. bursa-pastoris*	6.8 (0.2)	90.7 (0.2)	−2.5 (0.03)	—	4.8 (0.2) c	18.40	37
R- *M. aquaticum*	4.5 (10.4)	90.2 (10.3)	−2.1 (0.9)	—	5.0 (0.9) b	19.20	36

^a^R = biotypes of *C. bursa-pastoris* and *M. aquaticum*, target-site-resistant to tribenuron-methyl. ^b^y = C + (D-C)/{1+exp[b(log(x)-log (GR_50_))]}, where C is the lower limit of response, D is the upper limit of response, x is the herbicide rate, *y* is the percentage of dry weight residue, and b is the slope of the curve around the GR_50_. SE, standard error. ^c^The same letter were not significantly different at 0.05 level according to Fisher’s protected LSD test. ^d^SI calculated by SI = GR_10(Shannong 6)_/GR_90(weed)_.

### Field experiment

Visual estimation found no bleaching on any wheats during all the experiments in both years, and bipyrazone and its mixture with fluroxypyr-mepthyl were safe for wheats when applied POST at all the tested rates under field conditions (data not shown).

As shown in Table 5, although POST application of tribenuron-methy at 22.5 g a. i. ha^−1^ provided 90.7% population reduction against *D. sophia*, it showed low weed population reduction against *G. aparine* (53.1% population reduction) and failed to control *C. bursa-pastoris* with 8.9% weed population reduction at 20 DAT. Bipyrazone applied alone POST was very effective against *C. bursa-pastoris* and *D. sophia* but not *G. aparine* (weed population reduction <60%) at 20 DAT (Table 5). However, fluroxypyr-mepthyl had greater efficacy against *G. aparine* (weed population reduction å 97%) than against *C. bursa-pastoris* and *D. sophia* at 20 DAT (Table 5). The mixtures of bipyrazone and fluroxypyr-mepthyl were very effective against all tested weeds with a weed population reduction from 84.4% to 100% at 20 DAT (Table 5). Overall, the trend of weed population and biomass reduction at 40 DAT was similar to the weed population reduction at 20 DAT, and the control efficacy in 2018 was a little higher than in 2017 (Tables 5–7). Moreover, all herbicide treatments showed higher weed biomass reduction than weed population reduction at 40 DAT (Table 6 and Table 7).

**Table 5:** The weed population reduction of broadleaf weeds in wheat fields following different herbicide treatments at 20 days after treatment.

Treatment number	Weed population reduction (SE)^a^					
	2017			2018		
	*C. bursa-pastoris*	*D. sophia*	*G. aparine*	*C. bursa-pastoris*	*D. sophia*	*G. aparine*
	%			%		
1	87.9 (2.7) c	88.9 (3.9) b	36.5 (8.3) f	89.2 (2.5) c	89.7 (4.0) c	39.8 (6.2) e
2	90.3 (3.9) bc	90.7 (4.1) b	43.8 (4.6) ef	91.4 (2.6) c	93.1 (5.2) bc	44.7 (4.4) de
3	93.5 (3.0) abc	94.4 (3.8) ab	49.0 (13.0) de	95.7 (1.6) bc	96.6 (6.7) ab	51.5 (11.7) de
4	97.6 (1.7) ab	98.1 (3.3) a	55.2 (2.5) d	99.3 (1.4) a	98.3 (3.3) ab	58.3 (6.0) d
5	8.9 (6.2) e	90.7 (3.4) b	53.1 (3.3) de	9.4 (3.0) e	91.4 (9.9) bc	54.4 (5.9) d
6	54.0 (1.8) d	68.5 (6.3) c	97.9 (2.5) a	56.8 (2.6) d	62.1 (2.9) d	98.1 (2.5) a
7	94.4 (3.5) abc	95.1 (3.8) ab	84.4 (4.2) c	95.7 (1.7) bc	96.6 (4.2) abc	86.4 (5.4) c
8	95.2 (1.1) ab	95.7 (3.8) ab	89.6 (2.9) bc	96.4 (4.2) ab	96.6 (4.1) abc	91.3 (3.5) bc
9	98.4 (2.0) ab	98.7 (3.3) a	93.8 (2.6) b	99.3 (1.4) a	98.3 (3.6) ab	95.1 (6.1) ab
10	99.2 (1.5) a	99.4 (3.3) a	99.0 (2.1) a	100.0 a	100.0 a	99.0 (1.9) a

^a^Means followed by the same letter were not significantly different at 0.05 level according to Fisher’s protected LSD test. SE, standard error, n = 4.

**Table 6:** The weed population reduction of broadleaf weeds in wheat fields following different herbicide treatments at 40 days after treatment.

Treatment number	Weed population reduction (SE)^a^					
	2017			2018		
	*C. bursa-pastoris*	*D. sophia*	*G. aparine*	*C. bursa-pastoris*	*D. sophia*	*G. aparine*
	%			%		
1	86.3 (1.2) c	87.0 (3.1) b	34.4 (7.2) g	87.1 (1.9) d	84.5 (6.4) c	36.9 (15.4) f
2	88.7 (3.2) bc	89.1 (3.9) b	40.6 (8.1) fg	89.9 (0.5) cd	91.4 (4.4) bc	42.7 (10.5) ef
3	91.9 (4.0) abc	92.6 (0.7) ab	46.9 (12.9) ef	93.5 (6.5) bc	94.8 (3.5) ab	48.5 (4.8) de
4	96.0 (3.3) a	96.3 (4.2) a	53.1 (4.1) e	97.8 (1.4) ab	96.6 (4.3) ab	55.3 (10.8) d
5	4.8 (5.8) e	88.9 (4.3) b	51.0 (6.7) e	4.3 (3.4) f	89.7 (3.7) bc	52.4 (6.3) de
6	47.6 (0.6) d	57.4 (7.4) c	95.8 (3.6) ab	52.5 (2.9) e	58.6 (7.2) d	96.1 (3.0) a
7	92.7 (2.3) ab	92.8 (6.3) ab	82.3 (4.7) d	93.5 (3.4) bcd	94.8 (3.5) ab	84.5 (6.9) c
8	93.5 (0.9) ab	93.4 (0.7) ab	87.5 (3.8) cd	94.2 (3.6) bc	94.8 (6.5) ab	89.3 (7.7) bc
9	96.8 (2.5) a	96.6 (4.0) a	92.7 (2.0) bc	97.1 (4.2) ab	98.3 (3.1) a	93.2 (6.5) ab
10	97.6 (1.6) a	97.2 (4.4) a	97.9 (2.5) a	99.3 (1.4) a	98.3 (3.1) a	98.1 (2.5) a

^a^Means followed by the same letter were not significantly different at 0.05 level according to Fisher’s protected LSD test. SE, standard error, n = 4.

**Table 7:** The weed biomass reduction of broadleaf weeds in wheat fields following different herbicide treatments at 40 days after treatment.

Treatment number	Weed population reduction (SE)^a^					
	2017			2018		
	*C. bursa-pastoris*	*D. sophia*	*G. aparine*	*C. bursa-pastoris*	*D. sophia*	*G. aparine*
	%			%		
1	87.2 (1.6) c	88.1 (3.9) d	35.4 (7.5) f	88.0 (0.9) e	86.8 (6.1) d	38.0 (17.9) g
2	89.4 (3.3) bc	90.1 (3.8) cd	41.4 (7.6) ef	90.3 (0.7) de	91.9 (5.9) bcd	43.1 (11.2) fg
3	92.4 (3.7) abc	93.3 (0.9) cd	48.2 (13.2) de	94.0 (5.6) bcd	95.6 (3.0) abc	49.9 (3.6) ef
4	97.1 (2.1) a	97.2 (3.3) ab	54.1 (6.1) d	98.5 (1.2) abc	97.5 (3.8) ab	57.9 (11.4) e
5	9.0 (3.9) e	89.4 (4.3) cd	52.1 (7.6) d	8.3 (3.3) g	91.1 (3.0) cd	53.2 (9.6) ef
6	50.4 (1.7) d	61.0 (8.0) e	96.3 (3.1) a	58.8 (3.2) f	66.8 (7.3) e	97.1 (2.5) ab
7	93.1 (1.8) abc	94.2 (5.3) abc	83.1 (3.9) c	94.1 (3.7) cde	95.4 (3.4) abc	85.5 (7.7) d
8	94.1 (0.6) ab	94.6 (0.5) bcd	88.3 (2.9) bc	95.2 (3.2) bc	95.5 (5.3) abc	90.4 (8.3) cd
9	97.2 (2.2) a	97.3 (3.1) ab	93.2 (2.1) b	98.2 (2.2) ab	99.4 (1.1) a	94.4 (5.0) bc
10	98.1 (1.3) a	98.3 (2.0) a	98.6 (1.7) a	99.8 (0.5) a	99.3 (1.4) a	99.5 (0.7) a

^a^Means followed by the same letter were not significantly different at 0.05 level according to Fisher’s protected LSD test. SE, standard error, n = 4.

Wheat grain yields and yield growth rate following different treatments in 2017 and 2018 are presented in Table 8. With increased control of total weeds provided by the herbicide treatments, the grain yield growth rate was 2.2%-22.6% in 2017 and 2018 (Table 7 and Table 8). However, the grain yield was reduced by more than 23.5% by uncontrolled weed growth in the no treatment plots compared with hand weeding control treatment (Table 8). The wheat grain yields were higher in 2017 than in 2018 (Table 8).

**Table 8:** Wheat yields and yield growth rate following different herbicide treatments at Tai’an in 2017 and 2018.

Treatment number	2017		2018	
	Wheat grain yield (SE)^a^ (kg ha^−1^)	Yield growth rate (SE)^a^ (%)	Wheat grain yield (SE)^a^ (kg ha^−1^)	Yield growth rate (SE) (%)
1	7556 (115) fg	4.3 (1.6) fg	6694 (84) fg	4.8 (1.3) fg
2	7601 (136) ef	4.5 (1.9) ef	6728 (78) fg	5.3 (1.2) fg
3	7631 (75) ef	5.3 (1.0) ef	6803 (135) ef	6.5 (2.1) ef
4	7759 (78) e	7.1 (1.1) e	6953 (140) e	8.9 (2.2) e
5	7403 (86) gf	2.2 (1.2) g	6600 (65) g	3.3 (1.0) g
6	7609 (75) ef	5.0 (1.0) ef	6671 (70) fg	4.5 (1.1) fg
7	7980 (133) d	10.1 (1.8) d	7151 (124) d	12.0 (1.9) d
8	8209 (185) c	13.1 (2.5) c	7331 (111) c	14.8 (1.7) c
9	8576 (75) b	18.4 (1.0) b	7646 (112) b	19.7 (1.8) b
10	8715 (116) b	20.3 (1.6) b	7830 (91) a	22.6 (1.4) a
11	8946 (105) a	23.5 (1.4) a	7954 (171) a	24.5 (2.7) a
12	7245 (118) h	—	6386 (128) h	—

^a^Means followed by the same letter were not significantly different at 0.05 level according to Fisher’s protected LSD test. SE, standard error, n = 4.

## Discussion

Up to now, the most important chemical classes of commercial HPPD-inhibiting herbicides consist of isoxazoles such as isoxaflutole, pyrazoles such as topramezone, and triketones such as mesotrione, tembotrione, bicyclopyrone and sulcotrione^15,22^. Among HPPD-inhibiting herbicides, sulcotrione, a triketone derived from leptospermone from the bottlebrush plant, was the first to have its mode of action identified^23,24^. Meanwhile, triketone derivatives are most widely studied in China because its structure can be further divided into two parts, triketone and aromatic moieties^25–27^. Bipyrazone belongs to the same new chemical class of pyrazolones as topramezone^15^, presents the typical bleaching symptoms of an HPPD inhibitor after POST application.

Bipyrazone has the potential for control of broadleaf weeds such as *D. sophia*, *C. bursa-pastoris*, *L. arvense* and *M. aquaticum*, whereas it has little or no efficacy against grassy weeds like some HPPD-inhibiting herbicides, such as mesotrione^28^. According to the weed control spectrum experiment, the dry weight inhibition was over 85% for bipyrazone applied POST at 20 g a.i. ha^−1^ against all four broadleaf weed species above. Although the data obtained in the greenhouse may not be the same as that obtained in field conditions due to the complexity and changeability of field environments, it reflects the efficacy of weed control to a certain degree. For example, bipyrazone caused low dry weight inhibition of *G. aparine* at the tested rate (<20%) under greenhouse conditions, and consistently presented poor control efficacy (<60%) against *G. aparine* with POST application at all tested rates in field conditions. Study of wheat tolerance showed that bipyrazone POST application was safe for wheat. Also, the SI values for Shannong 6 wheat and five biotypes of three weed species were identified. The selectivity of the herbicide between the crop and the weeds rises with the increase of SI, and a herbicide can be safely used on a crop when the SI value is over 2.0^19,29^. Thus, bipyrazone applied POST was safe for Shannong 6 and effectively controlled *C. bursa-pastoris*, *M. aquaticum* and *D. sophia* with the SI value ranging from 34 to 39.

There was no difference in sensitivity to bipyrazone between susceptible and tribenuron-methyl resistant biotypes of *C. bursa-pastoris* and *M. aquaticum*. Nowadays, there are 326 biotypes of 76 wheat field weed species that have evolved resistance to herbicides with different modes of action such as acetolactate synthase (ALS) inhibitors and Acetyl-CoA carboxylase (ACCase) inhibitors, but there is still no report of HPPD-inhibitor resistant weeds in wheat fields^30^. With bipyrazone registration, there is a new mode of action being commercially introduced for the first time in China. Thus, bipyrazone may be an excellent tool to control some broadleaf weeds that have evolved resistance to herbicides with different modes of action. However, careful attention should be paid to preventing or delaying the development of resistance by reducing herbicide selection pressure via alternating between POST and preemergence (PRE), mixing herbicides with different modes of action, and rotation^31^. Also, increasing crop diversity and using biological control are practices to be encouraged^31^.

In the field experiment, bipyrazone applied POST alone were very effective against *C. bursa-pastoris* and *D. sophia* whereas it was not as effective against *G. aparine*. Although fluroxypyr-mepthyl showed excellent control of *G. aparine*, its effectiveness against *C. bursa-pastoris* and *D. sophia* was not satisfactory. The mixture of bipyrazone and fluroxypyr-mepthyl extended the weed-control spectrum and produced better control of all the weeds mentioned above. Combination of different herbicides not only extends the weed control spectrum but also delays the evolution of resistance better than herbicide rotation^31^. Tribenuron-methy failed to control *C. bursa-pastoris* in our field experiments, and the infestation of tribenuron-methy resistant *C. bursa-pastoris* has become a common and serious problem in Shandong and Henan Province, China^4,32^. Luckily, under field conditions, bipyrazone applied alone and its mixture with fluroxypyr-mepthyl also provided high efficacy against tribenuron-methy resistant *C. bursa-pastoris* as seen in the greenhouse study. The efficacy of all the treatments containing bipyrazone in 2018 was higher than 2017, which may result from the higher temperature 1 to 10 DAT (average temperature of 16.1 °C) in 2018 than 2017 (average temperature of 10.1 °C), which was consistent with the results from the greenhouse study that bipyrazone caused higher dry weight inhibition in 15/20 °C (night/day) than in 10/15 °C (night/day) (data not shown). Several studies have shown that temperature has great influence on herbicide efficacy^33–35^. It is reported that mesotrione, a HPPD-inhibiting herbicide widely used in maize fields, POST application showed different control efficacy at different temperature^36^. Temperature in the field may affect the efficiency of bipyrazone applied POST, and the application should be conducted when temperature is ideal for the control of sensitive weeds.

The higher grain yield rate obtained from using a mixture of bipyrazone and fluroxypyr-mepthyl, confirmed the greater total weeds control provided by the mixtures. Bipyrazone, tribenuron-methyl and fluroxypyr-mepthyl, when applied alone, resulted in unsatisfactory grain growth rates since the control of all broadleaf weeds was not warranted due to the limitation of weed control spectrum. In China, the weed flora of wheat fields is very complex; the broadleaf weeds are more common, but, the grassy weeds are spreading quickly^37^. This complexity makes it difficult to manage weeds successfully with a single herbicide. It is essential to expand the weed control spectrum by mixtures of two or three herbicides. Satisfactory yield growth rates were obtained with mixtures of bipyrazone and fluroxypyr-mepthyl at rates of 40 + 120 and 40 + 160 g a.i. ha^−1^ across years. Therefore, a mixture of bipyrazone and fluroxypyr-mepthyl (mass ratio = 1:3~4) applied POST at 160 to 200 g a.i. ha^−1^ is recommended for broad weed control in winter wheat fields, based on the results of our study. However, the choice of herbicides and application rates should depend on the actual weed flora in the fields. For example, 30 to 40 g a.i. ha^−1^ of bipyrazone applied alone can also be recommended when *C. bursa-pastoris* and *D. sophia* are the main weeds present.

Although hand weeding produced the highest yield growth rate in both years, it is uneconomical due to the labour and time costs^38,39^. The wheat yield in 2017 was higher than in 2018 because two episodes of heavy rain occurred in the test fields at the flowering stage in 2018 (daily precipitation> 78 mm, data not shown) which greatly reduced the yield.

In conclusion, results collected from both the greenhouse and field experiments suggest that bipyrazone, as a selective POST herbicide, has good potential for the management of broadleaf weeds including *D. sophia*, *C*. *bursa-pastoris*, *L. arvense* and *M. aquaticum* in winter wheat production systems in China. Especially with broadleaf weeds in wheat fields being rapidly evolving resistance to herbicides with different modes of action such as ALS inhibitors, protoporphyrinogen oxidase (PPO) inhibitors and synthetic auxins inhibitors^30^, bipyrazone, a new HPPD-inhibiting herbicide, may therefore be a powerful chemical broadleaf-weed-control technology, although it presents a limited weed control spectrum.

## Data Availability

All data generated or analysed during this study are available from the corresponding author on reasonable request.
